# Dynamic assembly of malate dehydrogenase-citrate synthase multienzyme complex in the mitochondria

**DOI:** 10.1101/2025.06.16.659985

**Published:** 2025-06-20

**Authors:** Joy Omini, Inga Krassovskaya, Taiwo Dele-Osibanjo, Connor Pedersen, Toshihiro Obata

**Affiliations:** 1, Department of Biochemistry and Center for Plant Science Innovation, University of Nebraska-Lincoln, 1901 Vine Street, Lincoln, Nebraska 68588, USA

## Abstract

The tricarboxylic acid (TCA) cycle enzymes, malate dehydrogenase (MDH1) and citrate synthase (CIT1), form a multienzyme complex called ‘metabolon’ that channels intermediate, oxaloacetate, between the reaction centers of the enzymes. Since the MDH1-CIT1 metabolon enhances the pathway reactions in vitro, it is postulated to regulate the TCA cycle flux through dynamic assembly in response to cellular metabolic demands. Here, we demonstrated that yeast mitochondrial MDH1 and CIT1 dissociated when aerobic respiration was suppressed by the Crabtree effect and associated when the pathway flux was enhanced by acetate. Pharmacological TCA cycle inhibitions dissociated the complex, while electron transport chain inhibition enhanced the interaction. The multienzyme complex assembly was related to the mitochondrial matrix acidification and oxidation, as well as cellular levels of malate, fumarate, and citrate. These factors significantly affected the MDH1-CIT1 complex affinity in vitro. Especially the buffer pH significantly changed the MDH1-CIT1 affinity within the pH range between 6.0 and 7.0, which is observed in the mitochondrial matrix under physiological conditions. These results show a dynamic association and dissociation of a metabolon in the mitochondria and its relationship with pathway flux, supporting the metabolon’s role in metabolic regulation. Multiple factors, including pH and metabolite availabilities, possibly regulate MDH1-CIT1 interaction.

## Introduction

Enzymes catalyzing sequential reactions can interact to form a multienzyme complex, often called a ‘metabolon,’ which channels the metabolic intermediate within the complex. Metabolite channeling can mediate pathway reactions by concentrating the reaction intermediates near the enzyme active site and sequestering them from competing reactions (Obata, 2020; Spivey and Ovádi, 1999; Wheeldon et al., 2016). Thus, dynamic multienzyme complexes are believed to quickly regulate cellular metabolic flux by changing their degree of association and dissociation without involving time-consuming and resource-demanding protein synthesis, degradation, and modification (Halper and Srere, 1977; Morgunov and Srere, 1998a; Obata, 2019; Omini et al., 2024; Srere, 1985; Sumegi et al., 1993). However, limited experimental evidence shows metabolon dynamics and the mechanisms regulating its association.

The tricarboxylic acid (TCA) cycle multienzyme complex composed of malate dehydrogenase (MDH) and citrate synthase (CS) is conserved in all organisms analyzed so far, including animals, bacteria, yeast, and plants (Meyer et al., 2011; Morgunov and Srere, 1998a; Robinson et al., 1987; Zhang et al., 2017). MDH and CS catalyze the key steps in respiratory metabolism as their reactions comprise the carbon entry steps from the glycolytic processes as malate and acetyl-CoA, respectively (Sweetlove et al., 2010). MDH-CS complex is considered a dynamic protein complex (Bulutoglu et al., 2016; Omini et al., 2021) that protects the channeled intermediate, oxaloacetate, from degradation and competing pathways in the bulk phase of the cell (Bulutoglu et al., 2016). Importantly, MDH-CS complex formation and oxaloacetate channeling are considered essential for the forward TCA cycle flux to occur since MDH forward reaction to synthesize oxaloacetate is thermodynamically unfavorable in physiological conditions (Fukuda et al., 2008). These findings suggest that dynamic association and dissociation of the MDH-CS multienzyme complex plays a role in the flux regulation of the TCA cycle and associated metabolic pathways. To address this hypothesis, this study focuses on demonstrating the in vivo dynamics of the MDH-CS complex in relation to respiratory fluxes, a crucial aspect of achieving metabolic regulation. Another key requirement, metabolic impacts of the metabolon, will be addressed in a separate study.

This study utilized the yeast mitochondrial MDH (MDH1)-CS (CIT1) multienzyme complex as the model. Budding yeast, *Saccharomyces cerevisiae*, dramatically rearranges its central carbon metabolism in response to changes in nutrient availability and stress conditions (Causton et al., 2001; Rep et al., 1999; Rolland et al., 2002). Remarkably, yeast respiratory metabolism, involving aerobic respiration and fermentation, is highly adaptable to substrate availability (Pfeiffer and Morley, 2014). When oxygen and non-fermentable respiratory substrates, such as acetate, are abundant, aerobic respiration is upregulated, with increased carbon flux through the TCA cycle (Gerstmeir et al., 2003; Lee et al., 2011). In the presence of fermentable sugars, such as glucose and fructose, the fermentation pathway is upregulated, and aerobic respiration is repressed, even when oxygen is available (Zhang et al., 2022). Furthermore, aerobic respiration and fermentation cooperate in the presence of a poorly fermentable carbon source like raffinose (Guaragnella et al., 2013). Application of fermentable substrates to respiring yeast cells induces a substantial shift from aerobic respiration to fermentation, known as the ‘Crabtree effect,’ involving a massive transcriptional rearrangement of enzyme genes related to aerobic respiration and fermentation (Gancedo, 1998; Ronne, 1995; Thierie and Penninckx, 2010). This inducible metabolic shift in yeast makes it an ideal model to investigate the dynamic relationship between the MDH1-CIT1 multienzyme complex and the TCA cycle flux. Understanding the mechanisms of the Crabtree effect is crucial for metabolic engineering applications to enhance the supply of TCA cycle intermediates for desired product synthesis (Yin et al., 2015) and for gaining insights into metabolic regulation in other eukaryotic systems, including cancer cells, which exhibit metabolic shifts similar to the Crabtree effect (Diaz-Ruiz et al., 2011).

Various allosteric regulators and environmental factors, including pH and redox state, affect MDH and CS enzyme activities (Beeckmans, 1984; Williamson and Cooper, 1980). These factors alter protein conformations, influencing the MDH-CS complex affinities. Previous in vitro studies have demonstrated that NAD^+^, malate, succinate, acetyl-CoA, α-ketoglutarate, and acidic pH enhance the MDH-CS interaction, while NADH, citrate, and basic pH weaken it (Omini et al., 2024, 2021; Tompa et al., 1987; Wu and Minteer, 2015). These allosteric regulators promote the MDH-CS interaction when the TCA cycle substrates are abundant and products are limited, indicating the role of the MDH-CS multienzyme complex in the TCA cycle feedback regulation. The electron transport chain activity affects the mitochondrial matrix pH, redox state, and ATP content and is closely related to cellular respiratory flux distributions (Santo-Domingo and Demaurex, 2012)). Therefore, the MDH-CS multienzyme complex may associate and dissociate according to the microenvironment in the mitochondrial matrix, such as metabolite concentrations, pH, redox state, and energy levels.

In this study, we adopted a nanoBIT protein-protein interaction assay system (Dixon et al., 2016) to monitor real-time MDH1-CIT1 multienzyme complex interaction in living yeast cells under the presence of various carbon sources and respiratory inhibitors. The results showed the relationship between respiratory flux and the dynamic MDH1-CIT1 complex assembly. The fluorescent biosensors showed crosstalk between the cellular respiratory status, MDH1-CIT1 interaction, and the microenvironments of the mitochondrial matrix. These results indicate the functional dynamics of the MDH-CS multienzyme complex in relation to the metabolic flux of the TCA cycle and adjacent pathways.

## Results

### MDH1-CIT1 interaction was detected exclusively in respiratory conditions.

**Figure 1.**
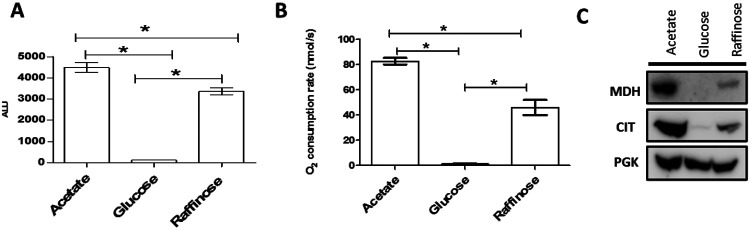
MDH1-CIT1 interaction under respiration, fermentation, and mixed-respiration conditions. Yeast cells were grown in the minimum media containing acetate (SD-Acet), glucose (SD-Gluc), and raffinose (SD-Raff) to the exponential growth phase. **(A)** Luciferase signal indicating MDH1-CIT1 complex interaction. **(B)** Cellular oxygen consumption rate. **(C)** MDH1 and CIT1 protein levels detected by Western blotting. In A-B, data are presented as mean ± s.d. and the differences between conditions were tested by Student’s *t*-test. Asterisks indicate significant differences with *p*<0.05.

We employed a nanoBIT split-luciferase system (Dixon et al., 2016) for semi-quantitative relative detection of the MDH1-CIT1 multienzyme complex association in living yeast cells. The codon-optimized sequences of nano-BIT subunits were integrated into the direct downstream of MDH1 and CIT1 exon in the yeast genome by gene homologous recombination to fuse the nanoBIT subunits to the native enzymes. The nanoBIT subunits reconstruct nanoLUC holoenzyme to exert luciferase signals when MDH1 and CIT1 are in proximity. The affinity between the nanoBIT subunits is very low and does not stabilize the interactions of the tagged proteins (Dixon et al., 2016). The integration of nanoBIT subunits showed no significant effect on the cellular growth rate or cellular MDH and CS enzyme activities (Figure 2 – figure supplement 1). Although the MDH1-CIT1 reporter line did not show detectable luciferase signal when it was grown in a fermentation condition (glucose-containing SD media; SD-Gluc), it showed substantial luciferase signal in mixed-respiration (raffinose-containing SD media; SD-Raff) and respiration (acetate-containing SD-media; SD-Acet) conditions (Figure 1A). These results indicate that MDH1 and CIT1 interact under respiratory conditions. The MDH1-CIT1 interaction can be related to the oxidative respiration and the TCA cycle activity since oxygen consumption was detected only in SD-Raff and SD-Acet media but not in the SD-Gluc medium (Figure 1B) as previously reported (Klein et al., 1998; Polakis and Bartley, 1965; Rep et al., 1999; Rolland et al., 2002; Strittmatter, 1957). However, MDH1 and CIT1 expression was downregulated in SD-Gluc growing cells near the detection limit (Figure 1C), making it difficult to conclude if the undetectable MDH1-CIT1 interaction was due to complex dissociation or low protein levels.

### Crabtree induction reduced the MDH1-CIT1 interaction.

**Figure 2.**
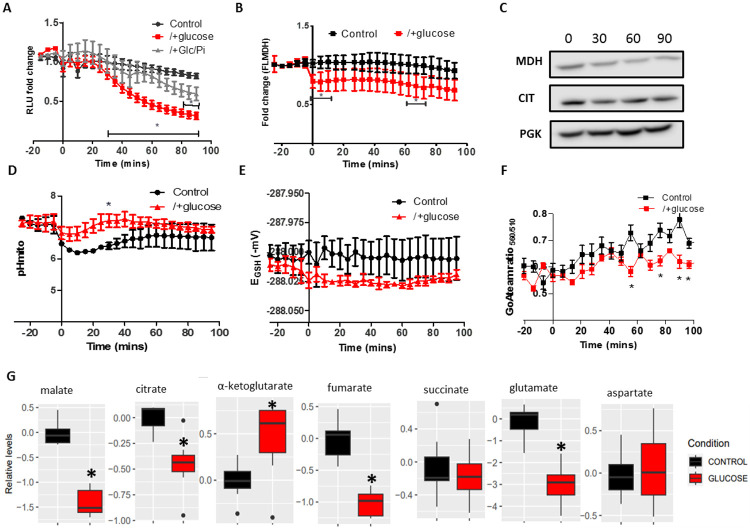
MDH1-CIT1 complex association, mitochondrial microenvironments, and cellular metabolite levels during Crabtree effect induction. Cells were cultured in fresh SD-Raff media in the control condition (black). The Crabtree effect was induced by the 2% glucose application to the SD-Raff-grown cells at 0 min (red). (A) NanoBIT signal indicating MDH1-CIT1 interaction. Relative luciferase unit (RLU) was calculated by normalizing the luciferase signals by the average signals during three pre-treatment time points. SD-Raff-grown cells were also co-treated with 2% glucose and a fermentation inhibitor, 100 mM phosphate, at 0 min (gray). (B) MDH1 protein levels monitored by the luminescence of MDH1 fused with full-length nanoLUC luciferase. (C) Western blot analysis of MDH1 and CIT1 protein levels after 0, 30, 60, and 90 min of Crabtree induction. Phosphoglycerate kinase (PGK) was detected as a loading control. (D) Mitochondrial matrix pH. (E) Mitochondrial matrix redox states as GSH/GSSG equivalent (mV). (F) Mitochondrial matrix ATP level indicated by the ratio between 560 and 510 nm emission signals. All data in A-F are presented as mean ± s.d. (G) Cellular metabolite levels at 80 min. The boxes, lines, error bars, and points indicate interquartile range, median, minimum, and maximum values, and outliers, respectively. Statistical differences against the control samples were assessed using the Student’s *t*-test at each time point. Asterisks indicate significant differences with *p*<0.05.

To test the relationship between the respiratory activity and the MDH1-CIT1 complex association when MDH1 and CIT1 enzymes are abundant in the cell, we monitored the time course of the MDH1-CIT1 interaction after a rapid shift from aerobic to anaerobic respiration by the Crabtree effect. The Crabtree effect was induced by applying 2% glucose to the SD-Raff-grown MDH1-CIT1 nanoBIT reporter line (Figure 2). The luciferase signals only slightly changed for 30 min upon glucose application, followed by a steep decline. In contrast, the control cells retained the initial signal level for 100 min (Figure 2A). Other Crabtree inducers, such as fructose and sucrose, also reduced the MDH1-CIT1 interaction, while the addition of non-fermentable sugars, including galactose, caused no significant change in MDH1-CIT1 interaction (Figure 2 – figure supplement 2A-D). The signal decline following glucose application was partially reversed by co-application with a fermentation inhibitor, 100 mM phosphate (van Urk et al., 1989; Figure 2A). Phosphate co-application slowed the decrease of luciferase signal compared to the glucose-applied cells, and the signal was not statistically significantly lower than the control until 80 min after application. The oxygen consumption rate significantly decreased when glucose was added to SD-Raff-grown cells but was slightly recovered by co-application of glucose with phosphate (Figure 2 – figure supplement 2E). These results indicate that the respiratory suppression by the Crabtree effect is related to MDH1-CIT1 complex disruption.

MDH1 protein level monitored with the yeast strain expressing MDH1 fused with full-length nanoLUC luciferase showed a 20% decline within 100 min upon glucose addition (Figure 2B). Western blotting also showed slight decrease in the MDH1 and CIT1 levels (Figure 2C). The time course of the MDH1-CIT1 nanoBIT signal was inconsistent with that of MDH1-nanoLUC, and the 20% decline in protein content cannot fully explain the decrease of MDH1-CIT1 signal by over 50% within 100 min after glucose addition (Figure 2A&B). Therefore, the decline in the MDH1-CIT1 nanoBIT signal reflects the MDH1-CIT1 complex dissociation co-occurring with the Crabtree effect, even though we cannot exclude a partial effect of protein degradation (Litsios et al., 2018).

Oxidative respiration significantly influences the microenvironments in the mitochondrial matrix (Beeckmans, 1984; Williamson and Cooper, 1980). To assess if the MDH1-CIT1 interaction decline is related to the changes in the microenvironments of the mitochondrial matrix by the Crabtree effect, we monitored the mitochondrial matrix pH, ATP concentration, and redox state by expressing fluorescent biomarkers, pHluorin, mito-GoAteam2, and mito-roGFP1, respectively, in the mitochondrial matrix of the MDH1-CIT1 reporter line (Figure 2 – figure supplement 3A-D). Mitochondrial matrix pH in the SD-Raff-grown cells was 7.2 and temporally declined to 6.8 in the first 25 min of glucose application. The control cell matrix pH gradually decreased to 6.2 and stayed lower than in glucose-applied cells (Figure 2D). The alterations of the MDH1-CIT1 interaction and mitochondrial matrix parameters in the control condition are not due to carbon starvation since 2% raffinose application had no significant effect (Figure 2 – figure supplement 3E). The redox state stayed around −288.0 mV in both the control and glucose cells (Figure 2E). The ATP level slightly increased during the experimental period of 100 min in both the control and glucose cells, with slightly but significantly lower levels in the glucose than control cells from 55 min to 100 min (Figure 2F). Thus, mitochondrial matrix pH and ATP levels were maintained after glucose was applied to cells, while they decreased and increased in the control cells during the experimental period.

Our previous in vitro study showed that metabolite abundance influences MDH-CS complex interaction (Omini et al., 2021). To assess the possible roles of metabolite accumulation in controlling MDH1-CIT1 complex interaction, we determined the cellular levels of 38 metabolites (Data S1) by gas chromatography-mass spectrometry (GC-MS). We focus here on the intermediates of the TCA cycle and related pathways (Figure 2G). At 80 min following 2% glucose application to SD-Raff-grown cells, malate, citrate, fumarate, and glutamate levels reduced while α-ketoglutarate content increased significantly compared to the control cells (Figure 2G). Thus, the glucose-induced shift from aerobic respiration to fermentation altered the intracellular metabolite profile in SD-Raff-grown cells.

### MDH1-CIT1 complex interacts in relation to the TCA cycle activity.

**Figure 3.**
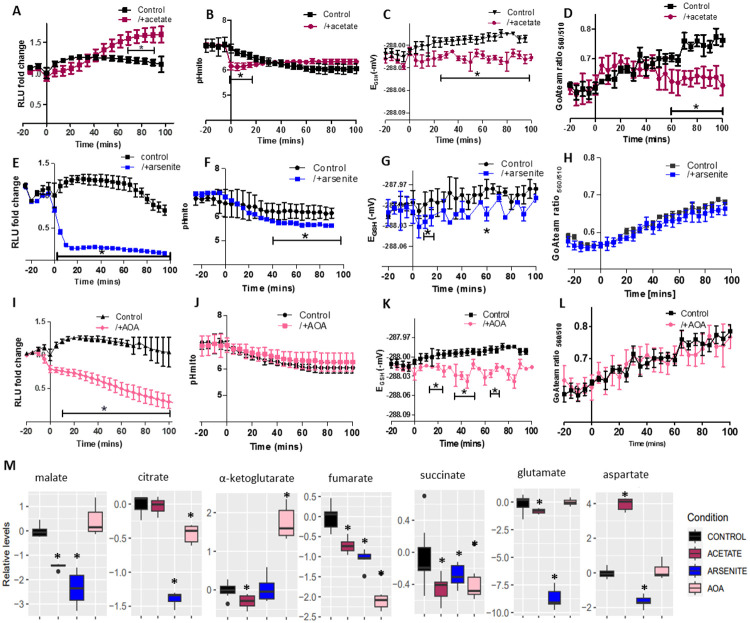
MDH1-CIT1 complex association, mitochondrial matrix microenvironments, and cellular metabolite levels following TCA cycle activation and inhibition. Cells were cultured in SD-Raff media in the control condition (black). The TCA cycle activator (acetate, dark red, **A-D**) and inhibitors (arsenite, blue, **E-H** and aminooxyacetate, pink, **I-L**) were applied at 0 min. **(A, E, I)** NanoBIT signal indicating MDH1-CIT1 interaction. Relative luciferase unit (RLU) was calculated by normalizing the luciferase signals by the average signals during three pre-treatment time points. **(B, F, J)** Mitochondrial matrix pH in control cells (black) and cells treated with acetate (dark red), arsenite (blue) and AOA (pink). **(C, G, K)** Mitochondrial matrix redox states as GSH/GSSG equivalent (mV). **(D, H, L)** Mitochondrial matrix ATP level indicated by the ratio between 560 and 510 nm emission signals of mito-GoATeam2 sensor. All data in A-L are presented as mean ± s.d. **(M)** Cellular metabolite levels after 80 min of treatment. The boxes, lines, error bars, and points indicate interquartile range, median, minimum, and maximum values, and outliers, respectively. Statistical differences against the control samples were assessed using the Student’s *t*-test at each time point. Asterisks indicate significant differences with *p*<0.05.

We further assessed the relationship between the MDH1-CIT1 complex association and TCA cycle flux. Acetate supplies acetyl-CoA to the TCA cycle and enhances the TCA cycle flux (Cavero et al., 2003). The application of 1% sodium acetate gradually increased the MDH1-CIT1 interaction (Figure 3A) with an immediate decline in mitochondria pH to 6.1 (Figure 3B). The mitochondrial matrix redox state and the ATP level were maintained during the experimental period while they gradually increased in the control cells, resulting in the significantly lower mitochondrial matrix redox state and ATP level in the acetate-treated cells in the latter half of the experimental period (Figure 3C&D). On the other hand, TCA cycle inhibition by 5 mM sodium arsenite, which impedes α-ketoglutarate dehydrogenase (Lee et al., 2011), led to an absolute decline in MDH1-CIT1 interaction within 10 min (Figure 3E). Mitochondrial matrix pH went slightly lower than the control cells after 40 min of arsenite application (Figure 3F). Arsenite treatment showed minor effects on the redox state and ATP level in the mitochondrial matrix (Figure 3G&H). Aminooxyacetate is an aminotransferase inhibitor and reduces TCA cycle flux by blocking the malate-aspartate NADH shuttle, which intersects with the cycle (Borst, 2020; Eto et al., 1999a; Molinié et al., 2022). The MDH1-CIT1 interaction gradually reduced following 0.5 mM aminooxyacetate application (Figure 3I). The matrix redox state was more reducing after 10 min of application (Figure 3K). Aminooxyacetate did not affect the mitochondrial matrix pH and ATP level (Figure 3J&L). Thus, the MDH1-CIT1 complex associates when the TCA cycle flux is activated and dissociates upon inhibition.

We evaluated the cellular metabolite profile after treatment of SD-Raff grown cells for 80 min with 1% acetate, 5mM arsenite, and 0.5 mM aminooxyacetate to determine the relationship between change in metabolite levels and the MDH1-CIT1 complex interaction (Figure 3M). Acetate application significantly reduced malate, α-ketoglutarate, fumarate, succinate, and glutamate levels, while aspartate abundance significantly increased. Inhibition of the TCA cycle by arsenite significantly reduced the levels of all TCA cycle metabolites other than α-ketoglutarate. Aspartate and glutamate were also decreased, and glutamate was almost depleted in the cell (Figure 3M). Aminooxyacetate significantly decreased citrate, fumarate, and succinate levels while it increased α-ketoglutarate abundance (Figure 3M).

### Electron transport chain inhibition enhanced the MDH1-CIT1 interaction, coinciding with matrix acidification and oxidation.

**Figure 4.**
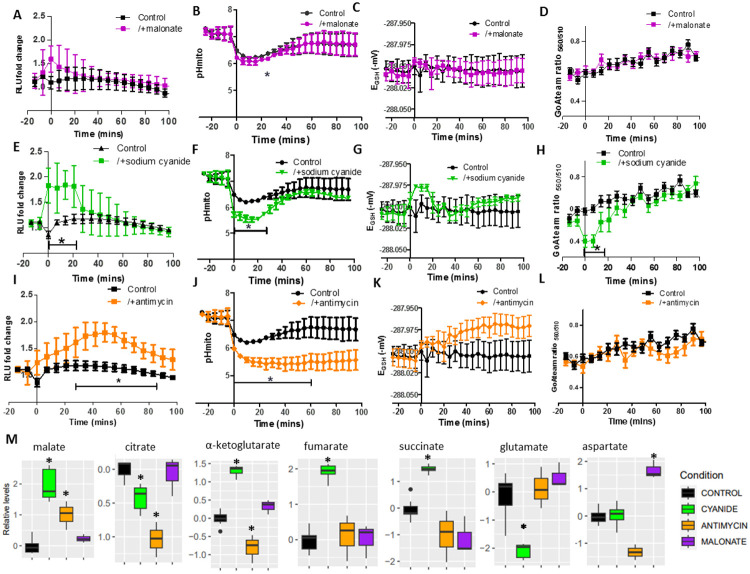
MDH1-CIT1 complex association, mitochondria microenvironments, and cellular metabolite levels following mitochondrial electron transport chain (ETC) inhibition. Cells were cultured in SD-Raff media in the control condition (black). The ETC inhibitors for complex II (malonate, purple, **A-D**), complex IV (cyanide, green, **E-H**), and complex III (antimycin, orange, **IL**) were applied at 0 min. **(A, E, I)** NanoBIT signal indicating MDH1-CIT1 interaction. Relative luciferase unit (RLU) was calculated by normalizing the luciferase signals by the average signals during three pre-treatment time points. **(B, F, J)** Mitochondrial matrix pH. **(C, G, K)** Mitochondrial matrix redox states as GSH/GSSG equivalent (mV). **(D, H, L)** Mitochondrial matrix ATP level indicated by the ratio between 560 and 510 nm emission signals of mito-GoATeam2 sensor. All data in A-L are presented as mean ± s.d. **(M)** Cellular metabolite levels after 30 min for malonate and cyanide and after 80 min for antimycin treatment. The boxes, lines, error bars, and points indicate interquartile range, median, minimum, and maximum values, and outliers, respectively. Statistical differences against the control samples were assessed using the Student’s *t*-test at each time point. Asterisks indicate significant differences with *p*<0.05.

Mitochondrial electron transport chain (ETC) activity is directly related to the mitochondrial microenvironments (Selivanov et al., 2008). To further assess the effects of mitochondrial matrix microenvironments on MDH1-CIT1 complex interaction, we treated SD-Raff-grown cells with ETC inhibitors. Inhibition of ETC complex II, III, IV, and V with 20 mM malonate, 10 μM antimycin, 0.5 mM cyanide, and 1 mM oligomycin, respectively, significantly reduced oxygen consumption of SD-Raff-grown cells (Figure 4 – figure supplement 1), showing their effects on respiration. MDH1-CIT1 interaction was enhanced by ETC inhibitors (Figure 4A, E, I), whereas the complex V (ATP synthase) inhibitor exerted no effect on MDH1-CIT1 interaction and mitochondrial matrix microenvironment in this experimental condition (Figure 4 – figure supplement 2A-D). Complex II inhibition slightly and temporally enhanced the MDH1-CIT1 interaction, although the increase was not statistically significant compared to the control (Figure 4A). Complex II inhibition showed minor effects on the mitochondrial microenvironments too (Figure 4B-D). The MDH1-CIT1 interaction was enhanced in the first 20 min of complex IV inhibition and reduced to the basal level afterward (Figure 4E). Mitochondrial matrix microenvironments were also affected in the first 20 min of Complex IV inhibition. The matrix pH and the ATP level were temporally decreased and exhibited inverse relationships with the MDH1-CIT1 interaction (Figure 4F&H). The mitochondrial matrix was slightly more oxidized during the initial 15 min of treatment, although the redox state was not significantly different from the control cells (Figure 4G). Complex III inhibition by antimycin slowly increased MDH1-CIT1 interaction, and the signal increase peaked at 60 min of treatment (Figure 4I). The mitochondrial matrix pH was reduced to 5.5 immediately after antimycin application and remained at the same level for 100 min (Figure 4J). The matrix was gradually oxidized following antimycin treatment, while the difference from the control cells was statistically insignificant (Figure 4K). The matrix ATP level was not affected by antimycin treatment (Figure 4L). These results indicate the relationship between MDH1-CIT1 interaction and mitochondrial matrix acidification and oxidation.

Cellular metabolite profiles were determined 30 min after malonate and cyanide treatment and 80 min after antimycin and oligomycin treatments. Complex II and V inhibition did not significantly alter cellular metabolite levels other than the aspartate accumulation in malonate-treated cells (Figure 4M, Figure 4 – figure supplement 5E-K). Complex IV inhibition by cyanide significantly reduced citrate and glutamate levels, while malate, alpha-ketoglutarate, fumarate, and succinate levels significantly increased (Figure 4M). Complex III inhibition significantly decreased citrate and α-ketoglutarate levels and increased the malate level (Figure 4M).

### MDH1-CIT1 interaction is affected by pH, redox state, ATP concentration, and metabolite availability in vitro.

**Figure 5.**
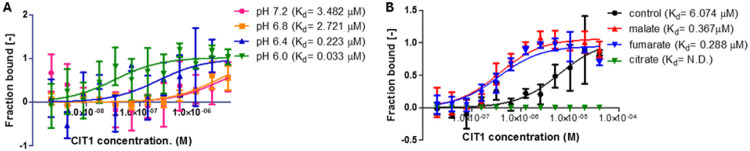
Effects of pH and metabolites on the yeast MDH1-CIT1 multienzyme complex affinity. The affinity of the MDH-CS multienzyme complex was analyzed by microscale thermophoresis (MST) using fluorescently labeled MDH1 as the target and CIT1 as the ligand. Curves represent the response (fraction bound) against CIT1 concentration. Data is presented as mean ± s.e.m. **(A)** Effects of pH. The MDH1-CIT1 interaction was determined in the buffer with pH 7.2 (pink), 6.8 (orange), 6.4 (olive green), 6.0 (green), and 5.8 (blue). **(B)** Effects of 10 mM malate (red), α-ketoglutarate (green), succinate (brown), citrate (blue), aspartate (purple), glutamate (pink), and fumarate (orange). The $K_{d}$ values of MDH1-CIT1 interaction were shown next to the legend.

To evaluate the specific effects of microenvironments and metabolite availability on the MDH1-CIT1 interaction, we investigated the MDH1-CIT1 affinity in vitro using recombinant proteins. Acidic pH significantly enhanced the binding affinity of the MDH1-CIT1 complex (Figure 5A) within the physiological range observed in this study (Figure 2D, Figure 3B, Figure 4B, F, J). The $K_{d}$ of the MDH1-CIT1 interaction was 3.48 μM at pH 7.2, while it was one magnitude lower at acidic pH (0.223 μM at pH 6.4 and 0.033 μM at pH 6.0). We also assessed the effects of the metabolites on the MDH1-CIT1 interaction (Figure 5B, Figure 5 – figure supplement 1). We tested malate, fumarate, citrate, α-ketoglutarate, succinate, glutamate, and aspartate since their cellular levels changed when the MDH1-CIT1 interaction altered (Figure 2G, Figure 3M, Figure 4M). Citrate destroyed the MDH1-CIT1 interaction, while malate and fumarate significantly enhanced complex affinity (Figure 5B). α-ketoglutarate, succinate, and aspartate slightly enhanced MDH1-CIT1 complex affinity, although glutamate did not affect the interaction (Figure 5 – figure supplement 1B). ATP also enhanced the MDH1-CIT1 complex affinity (Figure 5 – figure supplement 1A) as observed in porcine enzymes (Omini et al., 2021). Thus, malate, fumarate, citrate, ATP levels, and pH potentially influence the MDH1-CIT1 interaction in yeast mitochondria.

## Discussion

Here, we report the relationships between metabolic flux changes and mitochondrial MDH1-CIT1 multienzyme complex interaction in living yeast cells. MDH1-CIT1 multienzyme complex dynamically associated and dissociated in response to respiratory status. This study is the first in vivo evidence in any organism showing the dynamic association and dissociation of the TCA cycle metabolon in real time. The MDH-CS metabolon, specifically, is considered essential for the forward TCA cycle flux due to its ability to overcome the unfavorable thermodynamics of MDH reaction under physiological oxaloacetate concentrations (Beeckmans and Kanarek, 1981; Huang et al., 2018). The results of this study demonstrate that the MDH1-CIT1 multienzyme complex associates when the TCA cycle is active and dissociates when it is suppressed, supporting its hypothesized functions in metabolic regulation to enhance pathway flux by substrate channeling (Noor et al., 2014).

The MDH1-CIT1 complex dissociated under conditions suppressing the TCA cycle flux. TCA cycle inhibition by arsenite and aminooxyacetate induced strong dissociation of the MDH1-CIT1 complex (Figure 3E&I). In a physiological condition, the MDH1-CIT1 complex dissociated when Crabtree-inducing fermentable sugars were supplied to the media (Figs 1, 2, and S2). The Crabtree effect redistributes respiratory flux to stimulate fermentation and repress aerobic respiratory pathways, including the TCA cycle (Gancedo, 1998; Ronne, 1995). The metabolic shift by the Crabtree effect is a concerted action of multiple mechanisms, such as the suppression of enzyme gene expressions and the inhibition of mitochondrial transporters (Ahmadzadeh et al., 1996). Interestingly, glucose-induced rapid repression of mitochondrial respiration occurs at a rate that is too great to be explained only by the inhibition of enzyme synthesis, and additional forms of regulation are anticipated to be involved (Ahmadzadeh et al., 1996). Our results suggest that the dissociation of the TCA cycle metabolon can be an additional mechanism to downregulate the respiratory flux swiftly during the Crabtree effect.

On the other hand, the MDH1-CIT1 complex interaction was enhanced under conditions that promote TCA cycle flux. An increase in TCA cycle flux by the addition of acetate (Orlandi et al., 2014; Vilela-Moura et al., 2011) enhanced the MDH1-CIT1 complex interaction (Figure 3A), suggesting the role of the MDH-CS metabolon in facilitating forward TCA cycle flux. However, ETC inhibition also enhanced MDH1-CIT1 interaction (Figure 4A, E, and I) despite their inhibitory effects on oxidative respiration (Figure 4 - figure supplement 1) and the TCA cycle flux (Epstein et al., 2001; Grassian et al., 2014; Heinz et al., 2022). It should be noted that the enhanced MDH1-CIT1 interaction was temporal (Figure 4A, E, and I). It may not be directly related to the changes in respiratory metabolism as the inhibition of O_2_ consumption by ETC inhibitors was less significant than those by Crabtree induction (Figure 2 – figure supplement 2 and Figure 4 - figure supplement 1). The enhanced MDH1-CIT1 interaction is rather related to the temporal changes in mitochondrial microenvironments discussed below.

It should be noted that the treatments used to alter the TCA cycle flux in this study also induce flux alterations through metabolic pathways connected to the TCA cycle. In particular, acetate and aminooxyacetate treatments affect malate-aspartate shuttle activity (Cavero et al., 2003; Eto et al., 1999b; Liu and Butow, 2006), which competes for oxaloacetate with MDH and CS reactions. Since the protection of channeled intermediates from competing reactions is one of the postulated functions of metabolon (Morgunov and Srere, 1998b; Obata, 2019; Spivey and Merz, 1989; Welch, 1978), the relationship between malate-aspartate shuttle flux and MDH1-CIT1 interaction can provide significant insight into the metabolic function of MDH1-CIT1 metabolon. However, specific investigations are required to address this question since the TCA cycle and malate-aspartate shuttle function cooperatively, and their fluxes are tightly linked in living cells (Liao and Butow, 1993; Nielsen et al., 2011). Other metabolic pathways can also be affected by treatments, and their effects on MDH-CS interactions cannot be ruled out.

We also investigated the molecular mechanisms regulating the MDH1-CIT1 complex dynamics. We previously reported that protein conformation changes induced by the solution pH and allosteric regulators affect porcine MDH-CS complex interaction in vitro (Omini et al., 2021). The recombinant yeast MDH1 and CIT1 showed similar responses to the porcine enzymes; MDH1-CIT1 affinity was higher in low pH and in the presence of upstream TCA cycle intermediates (malate and fumarate) but disrupted in the presence of reaction product (citrate; Figure 5). These factors can affect the TCA cycle metabolon formation in vivo.

In this study, we observed enhanced MDH1-CIT1 interaction under conditions lowering mitochondrial matrix pH in vivo. The mitochondrial matrix pH decreased after adding acetate, corresponding to the enhanced MDH1-CIT1 interaction (Figure 3 A, B). The time courses of the temporal increase in MDH1-CIT1 interaction and pH decrease were inversely related following the complex III and IV inhibitions (Figure 4A, B, E, F). The decrease in the pH from 7.5 to 6.0 ranges is remarkable since the $K_{d}$ of MDH1-CIT1 association decreases in an order of magnitude within this pH range (Figure 5A). The mitochondrial matrix acidification under respiratory conditions likely favors the MDH1-CIT1 interaction. On the other hand, the mitochondria matrix pH was maintained around 7.2 in the presence of glucose (Figure 2D), which likely weakens the MDH1-CIT1 complex interaction. These results indicate that mitochondrial matrix pH, which is related to the proton transport activity by ETC, can stabilize or destabilize the MDH1-CIT1 complex to play a role in regulating the MDH1-CIT1 interaction in response to the respiratory activity.

The pH and MDH1-CIT1 interaction changes did not coincide following the Crabtree induction and acetate supplementation (Figs 2&3). TCA cycle inhibitions reduced the MDH1-CIT1 interaction without significantly affecting the mitochondrial matrix pH (Figure 3E, F, I, J). These results indicate that mitochondrial matrix pH is not always the primary regulator of MDH1-CIT1 interaction, which directly induces association and dissociation, but rather a factor maintaining the MDH1-CIT1 interaction state. Mitochondrial matrix oxidation can also influence the MDH1-CIT1 interaction state since it was related to the increase in MDH1-CIT1 interaction upon ETC inhibition (Figure 4G, K), while matrix reduction was related to the decrease in MDH1-CIT1 interaction upon aminooxyacetate-induced TCA cycle inhibition (Figure 3K).

The TCA cycle intermediates and cofactors can also regulate the MDH1-CIT1 interaction, considering their effects on the interaction in vitro (Omini et al., 2021). The yeast enzymes showed responses to malate, α-ketoglutarate, succinate, and citrate (Omini et al., 2021; Tompa et al., 1987; Wu et al., 2015) similar to the enzymes of other organisms, while fumarate is newly identified as an effector of the MDH-CS interaction. Especially fumarate, malate, and citrate showed significant influence on the yeast enzymes (Figure 5D). Cellular concentrations of these effector metabolites significantly altered under the conditions tested in this study (Figure 2G, Figure 3M, Figure 4M). Increased and decreased levels of malate and citrate, respectively, following complex IV and complex III inhibition (Figure 4M), likely enhance the MDH1-CIT1 interaction since citrate disrupts and malate enhances the interaction (Figure 5D; (Omini et al., 2021)). These results indicate the involvement of metabolite effectors, such as malate and citrate, in the MDH1-CIT1 interaction regulation. However, their precise effects must be evaluated by site-specific and time-dependent quantification of metabolite levels in the mitochondrial matrix.

**Figure 6.**
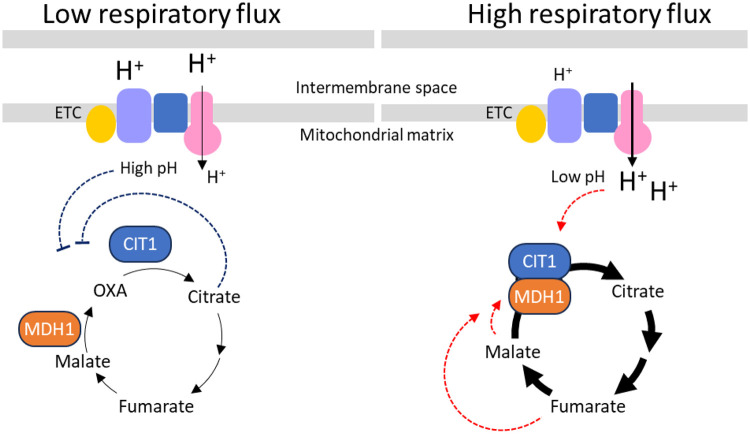
Relationship between the respiratory metabolism and the MDH1-CIT1 metabolon association. In conditions with low respiratory flux, the MDH1-CIT1 multienzyme complex dissociates, and the TCA cycle flux reduces. Reduced ETC flux results in higher mitochondrial matrix pH, which reduces MDH1-CIT1 affinity. When the respiratory flux and the TCA cycle flux are high, MDH1-CIT1 metabolon associates and likely channels the intermediate oxaloacetate (OXA). High ETC flux lowers mitochondrial matrix pH and enhances the MDH1-CIT1 interaction. The TCA cycle intermediates affect MDH1-CIT1 metabolon formation; fumarate and malate enhance (red arrows with dotted lines), and citrate inhibits (blue arrows with dotted lines) the interaction. The arrow thickness represents the metabolic fluxes.

This study demonstrates that the TCA cycle MDH1-CIT1 multienzyme complex dynamically interacts depending on the respiratory status of the cell. Cues of cellular respiratory state may be transmitted to the multienzyme complex, at least in part, by protons (pH), reducing equivalents (redox state), and metabolite levels (Figure 6). Since non of these factors always corresponded to the MDH1-CIT1 interaction, multiple factors most probably cooperatively regulate the multienzyme complex formation. Although we focused on allosteric regulators in this study, further factors are potentially involved in the MDH1-CIT1 complex regulation. For example, 44 and 33 post-translational modifications have been identified in CIT1 and MDH1, respectively (Bhagwat et al., 2021; Henriksen et al., 2012; Holt et al., 2009; Lanz et al., 2021; Reinders et al., 2007; Swaney et al., 2013; Weinert et al., 2013), some of which likely affect the MDH1-CIT1 complex affinity. Various scaffolding molecules, such as long noncoding RNAs, lipid layers, and scaffolding proteins, have been shown to stabilize the multienzyme complexes in other systems (Zhu et al., 2022). Future studies should investigate the effects of these factors to understand the regulatory mechanisms for MDH1-CIT1 interaction.

Changes in environmental conditions and nutrient availability occur swiftly, and a corresponding change in metabolic flux must occur at a similar rate for the successful adaptation of living cells. Dynamic metabolons can be a system to regulate and fine-tune metabolic network flux quickly, allowing cells to maintain metabolic homeostasis in rapidly fluctuating environments (Obata, 2019). Considering the thermodynamic favourability of forward MDH reaction and substrate channeling in vitro (Bulutoglu et al., 2016; Fukuda et al., 2008; Sweetlove et al., 2010), the MDH1-CIT1 complex formation likely enhances the forward TCA cycle flux in the conditions demanding oxidative respiration to achieve robust fine-tuning of the TCA cycle flux. Further direct evidence showing the effects of metabolon formation on the metabolic pathway flux in living cells is necessary to understand the functions of metabolons in metabolic network regulation. Elucidating the regulatory system of the TCA cycle metabolon can lead to a novel strategy to manipulate the TCA cycle flux in metabolic engineering to achieve efficient industrial production of various molecules requiring TCA cycle intermediates as substrates or to control the Warburg effect to suppress cancer cells.

## Materials and Methods

### Strains, media, and culture conditions

*Saccharomyces cerevisiae* BY4741 (MATa his3*Δ*1 leu2*Δ*0 met15*Δ*0 ura3*Δ*0) was used as the background strain. Cells were grown in synthetic complete medium (SD) containing 0.67% yeast nitrogen base lacking amino acids (Research Products International, Mt. Prospect, IL, USA) with 2% w/v D-raffinose and 1% amino acid complete drop-out mix. The complete amino acid drop-out mix was replaced with the amino acid drop-out mix lacking leucine for GoAteam expressing cells or the amino acid drop-out mix lacking uracil for RoGFP and pHluorin expressing cells. Cell growth cultures were incubated with shaking at 220 rpm at 28°C in an incubator shaker. Cell cultures were grown to exponential phase with an OD_600_=0.5 to 1.0 in all analyses.

### Oxygen consumption rate measurement

Oxygen consumption was measured using a Clark-type electrode (Oxygraph, Hansatech Instruments, Norfolk, UK) as described previously (Agrimi et al., 2011). Cells were grown to exponential phase OD_600_=0.5, harvested, centrifuged at 3,000 x *g* for 5 min at 4°C, and resuspended in growth medium to obtain a density of OD_600_=5.0. A total of 1 ml reaction volume consisting of equal parts of 445 μl cell culture, 445 μl growth media, and 10 μl of each inhibitor was added to the oxygraph chamber. Sodium malonate (20 mM), antimycin A (10 μM), sodium cyanide (0.5 mM), and oligomycin (1 mM) were used as specific inhibitors of electron transport chain complex II, III, IV, and V, respectively. The change in oxygen concentration was followed subsequently. Oxygen consumption rates were determined before and after the addition of each inhibitor from the slope of a plot of O_2_ concentration versus time. All measurements were conducted in triplicates.

### Generation of the *Saccharomyces cerevisiae* reporter strains with tagged MDH1 and CIT1

The nanoBiT split nanoLUC luciferase complementation system (Dixon et al., 2016) was adopted to monitor MDH1-CIT1 interaction in yeast cells. The yeast lines expressing MDH1 and CIT1 proteins fused with the small (SmBiT) and large (LgBiT) nanoBiT subunits, respectively, were generated by inserting the yeast codon-optimized tag-coding sequences into the BY4741 genome following the scarless C-terminal tagging procedure (Landgraf et al., 2016). The full-length nanoLUC, LgBiT, and SmBiT coding sequences were integrated to direct downstream of the Mdh1 (chrIV:3300230) and Cit1 (chrX:303993) genes on the BY4741 genome for C-terminal fusion. The LgBiT and SmBiT sequences include flexible linkers (ACKIPNDLKQKVMNH; (Hu et al., 2002)) with HA and cMyc epitope sequences, respectively, for immunological detections. Details of the vector construction procedure, primers, and vectors are described in the Supplementary Method.

Briefly, we generated the plasmids with integration cassettes composed of the linker, 5’ half of tag-coding sequence, URA3 selection marker, and 3’ half of tag-coding sequence in this order using the Golden Braid technology (Sarrion-Perdigones et al., 2013). The halves of the tag-coding sequences have an overlapping sequence. The integration cassettes were amplified by PCR using the primers with around 40 bases 5’ extension with sequences homologous to the franking region of the tag insertion sites. BY4741 cells were transformed with the purified PCR products to insert the cassettes into the target sites directly downstream of the Mdh1 and Cit1 genes by homologous recombination due to the 5’ extension of the primers. The transformants were selected on uracil-deficient plates. To pop-out the URA3 marker and reconstruct nanoLUC and nanoBIT subunits, the transformants were cultured overnight in uracil-containing YPD media. The URA3 gene was excluded from the genome by recombination based on the overlapping sequences in the cassette. The cell suspension was spread on SD plates containing 1 mg ml^−1^ 5-fluoroorotic acid (5-FOA) to select the cell lines without the URA3 gene. SmBiT was initially fused with the Mdh1 gene, and the resulting strain was further transformed to fuse LgBiT with Cit1 (MDH1/CIT1-BiT strain). Another strain expressing MDH1 fused with full-length nanoLUC luciferase was also generated following the same procedure to monitor MDH1 protein levels (MDH1-nLUC strain).

### In vivo MDH1-CIT1 interaction measurement

The MDH1/CIT1-BiT strain expressing MDH1 and CIT1 enzymes tagged with split halves of luciferase enzyme was grown to OD_600_ = 0.35-0.45. Cells were collected and resuspended to obtain a cell density of OD_600_=2.0 in fresh SD-Raff media. Each sample consisted of 80 μl of media, 10 μl of cells, and 10 μl of 50x furimazine luciferase substrate (Promega, Madison, WI, USA). The luminescence signal was measured every five min with a microplate reader (CLARIOSTAR Plus, BMG LABTECH, Ortenberg, Germany) at 28°C with 200 rpm shaking at the beginning of each cycle. Baseline luminescence was measured for 20 min before the treatment was applied. The time-dependent luminescence was measured for an additional 80 min after treatment.

### Western blotting

Cells were grown to OD_600_=0.5 and cell pellet was collected. Cells were first pretreated with 2 M LiAc and 0.4 M NaOH to permeabilize the cells and then treated with SDS-PAGE sample buffer to extract proteins according to the method described by Zhang et al. (Zhang et al., 2011). The cell lysate was centrifuged at 27,000 x *g* for 10 min at 4°C and the proteins were detected by SDS-PAGE and western blotting following the method described previously (Rajasekaran et al., 2022). cMyc Tag Monoclonal Antibody (MA1213161MG, Thermo Scientific) and HA Tag Monoclonal Antibody (LSG26183, Thermo Fisher Scientific) were used as primary antibodies to detect MDH1 and CIT1, respectively. PGK1 Monoclonal Antibody (Invitrogen 459250, Thermo Fisher Scientific) was used to detect phosphoglycerate kinase as a housekeeping protein and internal standard.

### In vitro MDH1-CIT1 interaction measurement

The recombinant MDH1 and CIT1 were produced accordingsuw to the method described in the Supplementary Methods. The interaction between recombinant MDH1 and CIT1 enzymes were analyzed by microscale thermophoresis (MST) according to the method described by Omini et al. (Omini et al., 2021) with slight modifications. Base MST buffer contained 50 mM Tris-HCL (pH 8), 150 mM NaCl, 10 mM MgCl_2_, 5 mM DTT, and 0.05% Tween-20. Recombinant MDH1 (10 μM) was labeled with Protein Labeling Kit RED-NHS 2nd Generation (NanoTemper, München, Germany) and used as the target. CIT1 was used as the ligand. Two-times serial dilution of 80 μM CIT1 was conducted for 16 concentrations. A total of 10 μL of CIT1, 10 μL MST buffer, and 10 μL labeled MDH1 were mixed and loaded to Monolith NT.115 Capillaries (NanoTemper). Capillaries were incubated at room temperature for 1 min, and the interaction was analyzed by MST using Monolith NT.115 (NanoTemper). To test the effect of pH on MDH1-CIT1 interaction, 50 mM tris buffers with pH 6.0, 7.5, and 8.0 were prepared and used as the MST buffer for sample preparation. To test the effect of reducing environment on interaction, various concentrations (5 mM, 2.5 mM, 1.25 mM) of DTT were prepared with the MST buffer and used for sample preparation. To test the effect of ATP level on interaction, an MST buffer containing different concentrations of ATP (5 mM, 2.5 mM, 1.25 mM) was made and used for sample preparation. To test the effect of metabolite availability on the interaction, MST buffer containing respective metabolites at 10 mM concentration was made and used for sample preparation and experiments.

### Enzyme activity assays

Cells were grown to an exponential phase with OD_600_ of 0.5, and 2 ml of the cells were harvested for enzyme activity assay. Yeast cell lysates were prepared by disruption with glass beads as described previously (Mukherjee et al., 2020), with the lysis buffer omitted butylhydroxytoluene. The protein concentration of the lysate was determined using Pierce^™^ BCA Protein Assay Kit (Thermo Fischer Scientific). CS activity was determined using a method described previously (Srere, 1969) that measures free thiols by coupling the citrate synthase reaction to thiol reaction with 5, 5’-dithiobis-(2-nitrobenzoate) (DTNB). The CS enzyme activity assay mixture contained 154 μL of distilled water, 20 μL of 1 mM DTNB, and 6 μL of 10 mM acetyl CoA. The citrate synthase reaction was initiated by the addition of 10 μL of 10 mM oxaloacetate. The absorption at 412 nm was followed to measure citrate synthase activity. MDH activity assay mixture contained 50 mM TES (pH 7.2), 5 mM MgCl_2_, 0.2 mM NADH, and 0.05% Triton X100. To obtain a total reaction volume of 300 μL, 285 μL of the assay mixture and 5 μL cell lysate were added to the wells, and the reaction was initiated with 10 μL of 30 mM oxaloacetate. Reduction of NADH was followed at 340 nm to determine MDH enzyme activity. Enzyme activity was measured using a microplate reader absorbance function (CLARIOSTAR Plus, BMG LABTECH).

### Metabolite profiling

Yeast cells were grown to the exponential phase (OD_600_~0.5), and treatments were applied and incubated for the time described in the figure legends. Cellular metabolites were extracted following the protocol described by Obata et al. (Obata et al., 2013). The cells in 1 mL culture were harvested by vacuum filtration using a membrane filter (0.45 μm HV Durapore 25 mm diameter; MilliporeSigma, Burlington, MA, USA). The filter was put into a 2 mL microcentrifuge tube, flash-frozen in liquid nitrogen, and stored at −80°C. The metabolites were extracted from the filtered cells with methanol: water: chloroform, and 50 μL aliquot was dried down by vacuum centrifugation. Dried metabolites were derivatized with methoxyamine hydrochloride in pyridine and further trimethylsylilated by N-Methyl-N-(trimethylsilyl) trifluoroacetamide (MilliporeSigma). Derivatized samples were analyzed by 7200 GC-QTOF system (Agilent, Santa Clara, CA, USA) exactly as described in Wase et al. (Wase et al., 2022). Each metabolite’s peak height was normalized by the peak height of the internal standard (ribitol) to represent relative levels of metabolite.

### Expression of mitochondrial biosensors

The pH, redox state, and ATP levels in the mitochondrial matrix were measured using the mito-Go Ateam2, mito-ROGFP1, and pHluorin (pAG416-COX4-pHluorin, URA selection marker) fluorescence biosensors, respectively, specifically localizing in the mitochondrial matrix. The mito-Go Ateam2 (p415-GPDpro-mito GO ATeam) and mito-roGFP1 (p416-GPDpro-mito roGFP) encoding plasmids (Vevea et al., 2013a) were generous gifts from Dr. Liza Pon at Department of Pathology and Cell Biology, Columbia University. The pHluorin encoded plasmid (pAG416-COX4-pHluorin;(Ayer et al., 2013)) was a generous gift from Dr. Anita Ayer at the University of New South Wales, Sydney, Australia. The plasmids encoding these biosensors were transformed into split-Luc tagged MDH1/CIT1 strain using the lithium acetate method (Chen et al., 1992). The p416-GPDpro-mito roGFP and pAG416-COX4-pHluorin harboring cells were selected on URA-media, and the cells with p415-GPDpro-mito GO ATeam were selected on Leu-media.

The localization of biosensors within the mitochondria was confirmed by confocal microscopy. Cells were grown in SD selection media to OD_600_=0.50. Cells were stained with MitoTracker Deep Red FM (ThermoFisher Scientific) in a 100 nM dye solution for 30 min with shaking at 28 °C to visualize the mitochondria. The stained cells were resuspended in fresh 10 mM HEPES buffer (pH 7.4) with 2% raffinose to obtain a final concentration of OD_600_=10. Confocal imaging was performed on an A1R-Ti2 Confocal Laser Scanning Microscope (Nikon, Tokyo, Japan) with a Plan Apo 60x 1.40 Oil lens 0.17 WD 0.13 (Nikon) and a 2x digital zoom for a total magnification of 1200x. Brightfield and fluorescent images were captured. Imaging was done sequentially with excitation at 405 nm and emission at 425-475 nm for pHluorin and mito-roGFP1, excitation at 488 nm and emission at 500-550 nm for mito-GoAteam2, and excitation at 640 nm and emission at 663-738 nm for MitoTracker. Images were collected with NIS Elements software (Nikon) and processed with ImageJ analysis software (Vevea et al., 2013b).

### Measurement of redox state, pH, and ATP level in the mitochondrial matrix

Cells expressing pHluorin, mito-roGFP1, and mito-GoAteam2 were grown to OD_600_=0.50 in their respective selection media. Cells were prepared for fluorescence measurement according to the method described by Morgan *et al* (Morgan et al., 2011). Time-based fluorescence intensity was measured using a microplate reader (CLARIOSTAR Plus, BMG LABTECH) at 28°C with shaking at 200 rpm with 20 min of baseline measurement before treatments. In all experiments, the strains harboring empty vectors were grown simultaneously as a reference for background fluorescence at the different excitation wavelengths. Background fluorescence was subtracted from fluorescence intensity from cells expressing biosensors.

For the pH measurement, the ratio of emission intensity at 510 nm resulting from excitation of pHluorin at 390 nm and 470 nm was calculated (R390/470) using pHluorin expressing strain. The mitochondrial matrix pH was calculated from the R390/470 ratio using a calibration curve generated according to the method described by Orij *et al* (Orij et al., 2009). Briefly, pHluorin-expressing cells were permeabilized by resuspension in PBS containing 100 μg ml-1 digitonin for 10 min and washed with PBS. Cells were resuspended in citric acid/Na2HPO4 buffer of pH values ranging from 5.0 to 9.0. The ratio of pHluorin emission at 510 nm upon excitation at 390 and 470 nm (R390/470) was plotted against buffer pH to obtain a calibration curve.

The mitochondrial matrix redox state was measured using mito-roGFP1 expressing strain. The ratio of emission intensity at 510 nm from excitation at 365 nm and 470 nm (R470/365) was measured. Mito-roGFP1 in situ calibration was performed following the method described by (Vevea et al., 2013b). Digitonin-treated mito-roGFP1 expressing cells were incubated in 0, 5, and 10 mM H_2_O_2_ and DTT for 20 min at 28°C with shaking at 200 rpm. The R470/365 was plotted against the redox potential of the solutions calculated using the formula described previously (Morgan et al., 2011).

Relative ATP level was determined by measuring the ratio of the emission intensity of mito-GoAteam2 at 510 nm and 560 nm with excitation at 470 nm (R560/510). The ATP levels were analyzed as the relative value without calibration to absolute concentrations.

### Statistical Analysis.

The differences between the control and test samples were evaluated by a two-tailed unpaired Student’s *t*-test. *p* < 0.05 was considered a statistically significant difference. For the time course data, the test was applied at each time point. All data was obtained from triplicated independent experiments.

## Supplementary Material

1 **Figure 2 – figure supplement 1.** Growth rate and enzyme activity of nanoBIT reporter strain.
**Figure 2 – figure supplement 2.** Effects of sugars on MDH1-CIT1 complex assembly and oxygen consumption rate.
**Figure 2 – figure supplement 3.** Biosensors indicate mitochondria microenvironments.
**Figure 4 - figure supplement 1.** Effect of ETC inhibitors on O_2_ consumption rate.
**Figure 4 – figure supplement 2.** Effects of Complex V inhibition on MDH1-CIT1 complex association, mitochondrial microenvironments, and cellular metabolite levels.
**Figure 5 – figure supplement 1.** Effects of metabolites and ATP on the yeast MDH1-CIT1 multienzyme complex affinity.
