# Supplementary material for: Dynamic assembly of malate dehydrogenase-citrate synthase multienzyme complex in the mitochondria
Source: bioRxiv. 2025 Jun 20:2025.06.16.659985. Preprint. [Version 1] doi: 10.1101/2025.06.16.659985 (PMC12262645; doi:10.1101/2025.06.16.659985)
Supplement: 1 — Figure 2 – figure supplement 1. Growth rate and enzyme activity of nanoBIT reporter strain. Figure 2 – figure supplement 2. Effects of sugars on MDH1-CIT1 complex assembly and oxygen consumption rate. Figure 2 – figure supplement 3. Biosensors indicate mitochondria microenvironments. Figure 4 - figure supplement 1. Effect of ETC inhibitors on O2 consumption rate. Figure 4 – figure supplement 2. Effects of Complex V inhibition on MDH1-CIT1 complex association, mitochondrial microenvironments, and cellular metabolite levels. Figure 5 – figure supplement 1. Effects of metabolites and ATP on the yeast MDH1-CIT1 multienzyme complex affinity. [file NIHPP2025.06.16.659985v1-supplement-1.pdf]

bioRxiv preprint doi: https://doi.org/10.1101/2025.06.16.659985; this version posted June 20, 2025. The copyright holder for this preprint (which was not certified by peer review) is the author/funder, who has granted bioRxiv a license to display the preprint in perpetuity. It is made available under aCC-BY 4.0 International license.

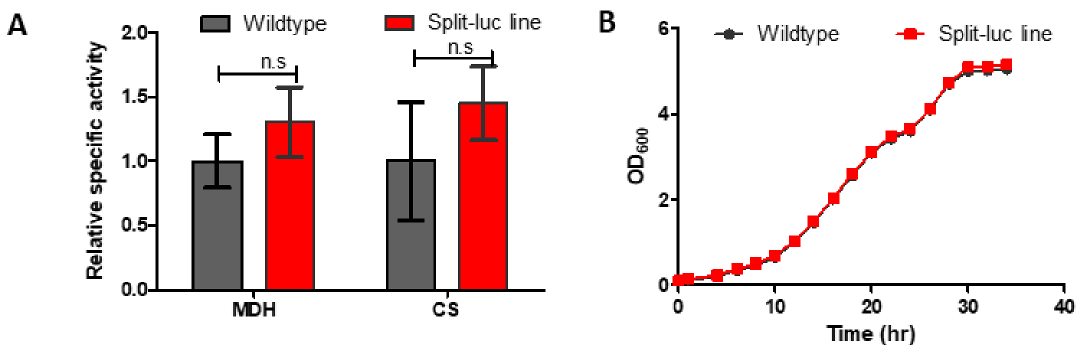

Figure 2 – figure supplement 1. Growth rate and enzyme activity of nanoBIT reporter strain. (A) Extractable cellular MDH and CS enzyme activities in the wildtype (black) and nanoBIT reporter strain (Split-luc line, red). (B) Cellular growth of cells in SD-raff media monitored as culture OD_{600}. Data is presented as mean ± s.d. Statistical differences against the wildtype samples were assessed by Student's *t*-test at each time point. n.s., not significant (p>0.05).

bioRxiv preprint doi: https://doi.org/10.1101/2025.06.16.659985; this version posted June 20, 2025. The copyright holder for this preprint (which was not certified by peer review) is the author/funder, who has granted bioRxiv a license to display the preprint in perpetuity. It is made available under aCC-BY 4.0 International license.

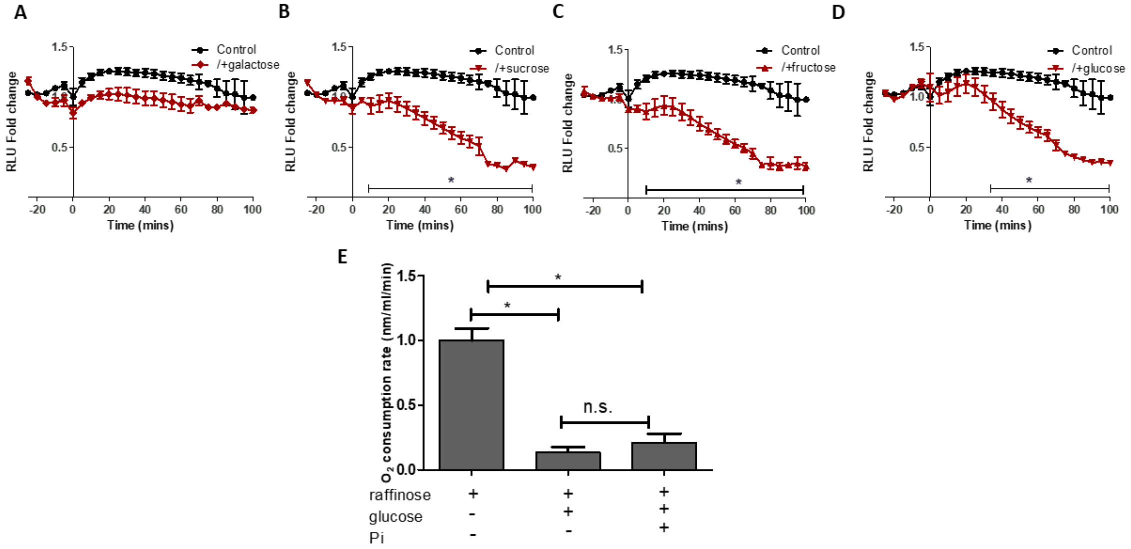

Figure 2 – figure supplement 2. Effects of sugars on MDH1-CIT1 complex assembly and oxygen consumption rate. (A-D) NanoBIT signal indicating effect of galactose, sucrose, fructose, and glucose on MDH1-CIT1 interaction. Cells were cultured in fresh SD-Raff media in the control condition (black). The cells are treated with 2% sugar application to the SD-Raff-grown cells at 0 min (red). Relative luciferase unit (RLU) was calculated by normalizing the luciferase signals by the average signals during three pre-treatment time points. (E) Effects of glucose and inorganic phosphate (fermentation inhibitor) on oxygen consumption rate. Basal O_2 consumption rate of SD-Raff grown cells was measured. Glucose and inorganic phosphate were added and O_2 consumption rate was measured for 5 minutes. All data in A-E are presented as mean ± s.d. Statistical differences against the control samples were assessed by Student's *t*-test at each time point. Asterisks indicate significant differences with p<0.05.

bioRxiv preprint doi: https://doi.org/10.1101/2025.06.16.659985; this version posted June 20, 2025. The copyright holder for this preprint (which was not certified by peer review) is the author/funder, who has granted bioRxiv a license to display the preprint in perpetuity. It is made available under aCC-BY 4.0 International license.

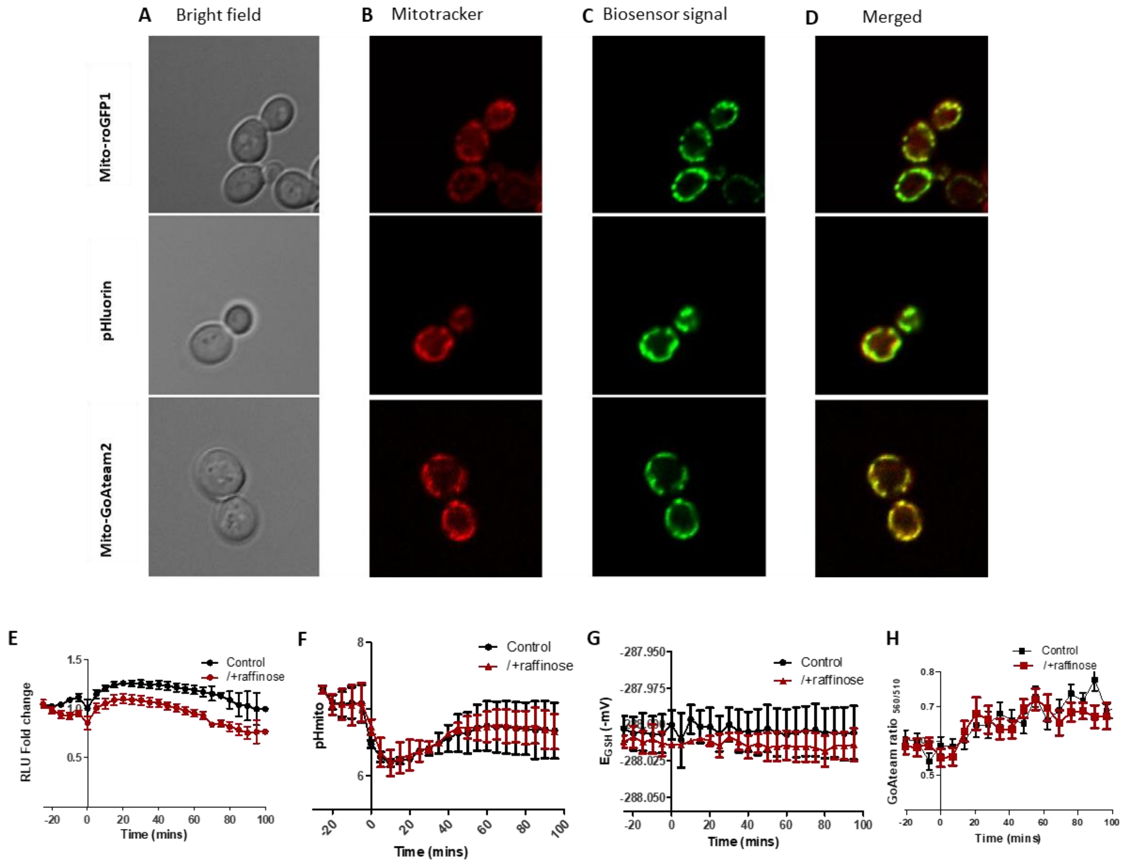

Figure 2 – figure supplement 3. Biosensors indicate mitochondria microenvironments. (A-D) Subcellular localizations of fluorescent biosensors. The yeast strains expressing Mito-roGFP1 (upper panels), pHluorin (middle panels), and Mito-GoATeam2 (lower panels) were observed by a fluorescent microscopy in the SD-Raff media. The cells were stained with Mitotracker orange prior to the analysis. (A) Bright field image. (B) Mito tracker signal. (C) Biosensor signals. (D) Merged images of the A to C. (E) Effect of raffinose addition on MDH1-CIT1 interaction. Cells were cultured in SD-Raff media in the control condition (black). The cells are applied with 2% raffinose to the SD-Raff-grown cells at 0 min (red). Relative luciferase unit (RLU) was calculated by normalizing the luciferase signals by the average signals during three pre-treatment time points. (F) Mitochondria matrix pH in control cells (black) and cells applied with additional raffinose (red). (G) Mitochondrial matrix redox state reported as redox potential of roGFP1 (mV). (H) Mitochondrial matrix ATP level indicated by the ratio between 560 and 510 nm emission signals of mito-GoATeam2 sensor. Data in E-H are presented as mean ± s.d.

bioRxiv preprint doi: https://doi.org/10.1101/2025.06.16.659985; this version posted June 20, 2025. The copyright holder for this preprint (which was not certified by peer review) is the author/funder, who has granted bioRxiv a license to display the preprint in perpetuity. It is made available under aCC-BY 4.0 International license.

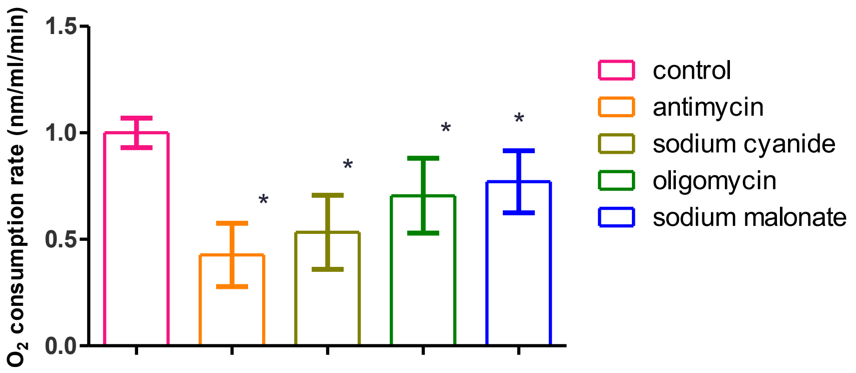

Figure 4 - figure supplement 1. Effect of ETC inhibitors on O_2 consumption rate. Basal O_2 consumption rate was measured, then inhibitor was added and O_2 consumption rate was measured for 5 minutes. Data is presented as mean ± s.d. Statistical differences against the control samples were assessed by Student's *t*-test. Asterisks indicate significant differences with $p<0.05$.

bioRxiv preprint doi: https://doi.org/10.1101/2025.06.16.659985; this version posted June 20, 2025. The copyright holder for this preprint (which was not certified by peer review) is the author/funder, who has granted bioRxiv a license to display the preprint in perpetuity. It is made available under aCC-BY 4.0 International license.

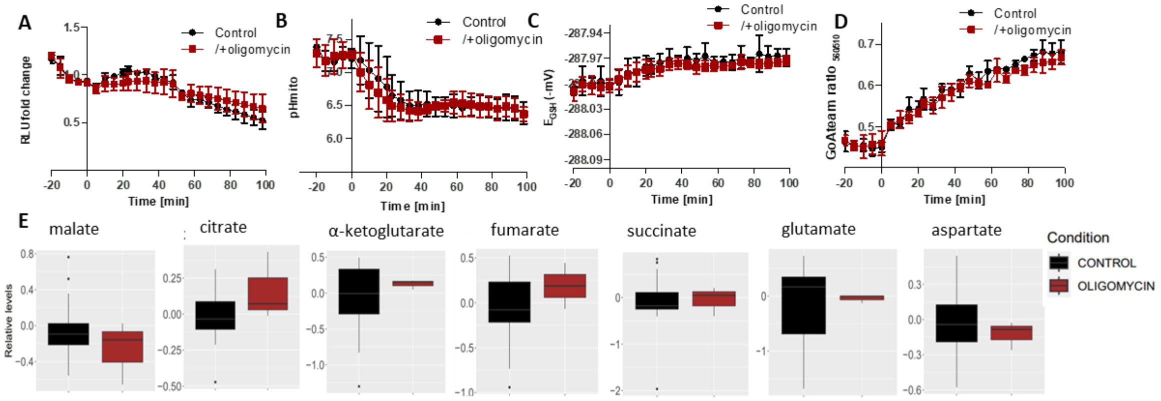

Figure 4 – figure supplement 2. Effects of Complex V inhibition on MDH1-CIT1 complex association, mitochondrial microenvironments, and cellular metabolite levels. Cells were cultured in SD-Raff media in the control condition (black). Oligomycin was applied at 0 min (red). (A) NanoBIT signal indicating MDH1-CIT1 interaction. Relative luciferase unit (RLU) was calculated by normalizing the luciferase signals by the average signals during three pre-treatment time points. (B) Mitochondrial matrix pH. (C) Mitochondrial matrix redox states as GSH/GSSG equivalent (mV). (D) Mitochondrial matrix ATP level indicated by the ratio between 560 and 510 nm emission signals of mito-GoATeam2 sensor. All data in A-D are presented as mean ± s.d. (E) Cellular metabolite levels after 80 min of oligomycin treatment. The boxes, lines, error bars, and points indicate interquartile range, median, minimum, and maximum values, and outliers, respectively. Statistical differences against the control samples were assessed by Student's *t*-test at each time point. Asterisks indicate significant differences with $p<0.05$.

bioRxiv preprint doi: https://doi.org/10.1101/2025.06.16.659985; this version posted June 20, 2025. The copyright holder for this preprint (which was not certified by peer review) is the author/funder, who has granted bioRxiv a license to display the preprint in perpetuity. It is made available under aCC-BY 4.0 International license.

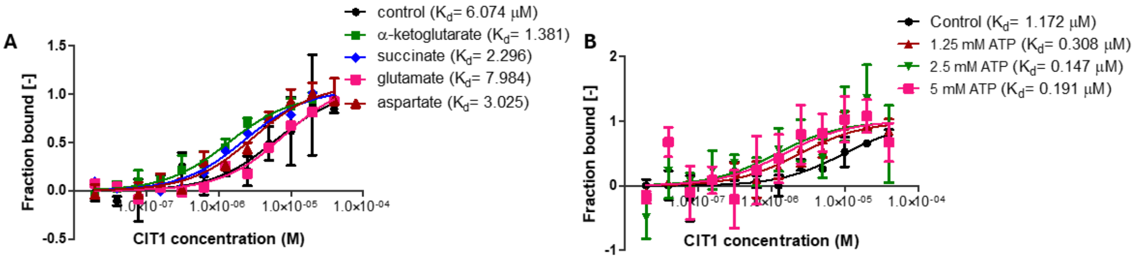

Figure 5 – figure supplement 1. Effects of metabolites and ATP on the yeast MDH1-CIT1 multienzyme complex affinity. The affinity of the MDH1-CIT1 multienzyme complex was analyzed by microscale thermophoresis (MST) using fluorescently labeled MDH1 as the target and CIT1 as the ligand. Curves represent the response (fraction bound) against CIT1 concentration. Points represent the means of fraction bound, and the error bars represent the standard deviations of three measurements. (A) Effects of metabolites. The MDH1-CIT1 interaction was determined in the buffer (control; black) with 10 mM α-ketoglutarate (green), 10 mM succinate (blue), 10 mM glutamate (pink), and 10 mM aspartate (dark red). (B) Effects of 1.25 mM (brown), 2.5 mM (green), and 5 mM (pink) ATP. The K_d values of MDH1-CIT1 interaction were shown next to the legend.

bioRxiv preprint doi: https://doi.org/10.1101/2025.06.16.659985; this version posted June 20, 2025. The copyright holder for this preprint (which was not certified by peer review) is the author/funder, who has granted bioRxiv a license to display the preprint in perpetuity. It is made available under aCC-BY 4.0 International license.

Supplementary Dataset 1 (separate file). Metabolite profiling data of the *S. cerevisiae* cells. The relative metabolite levels were used for the analyses in this study. The raw peak heights, quantitative ion m/z, and the retention time of the each analyzed peak are also indicated. The metabolite profiling was conducted in two batchs. CONTROL1, GLUCOSE, CYANIDE, ACETATE, ARSENITE, and AOA conditions were analyzed in the first batch (n=7). CONTROL2, MALONATE, OLIGOMYCIN, and ANTIMYCIN were analyzed in the second batch (n=3).
